# Small Non-Coding RNAs in Leukemia

**DOI:** 10.3390/cancers14030509

**Published:** 2022-01-20

**Authors:** Veronica Balatti, Carlo M. Croce

**Affiliations:** 1 Department of Cancer Biology and Medical Genetics, The Ohio State University, Columbus, OH 43210, USA; 2 The Ohio State University Comprehensive Cancer Center, The Ohio State University, Columbus, OH 43210, USA

**Keywords:** leukemia, small non-coding RNAs, microRNA, miRNA, tRNA fragments

## Abstract

**Simple Summary:**

Leukemia is a group of blood cancers that arise when abnormal or underdeveloped blood cells accumulate in the bloodstream, bone marrow and lymphatic system. A driver event in the development of Chronic Lymphocytic Leukemia (CLL) is the deletion of the genomic region encoding for *miR-15a/16-1.* MicroRNAs are small non-coding RNAs involved in the regulation of gene expression, and loss of *miR-15a/16-1* in CLL results in the accumulation of the antiapoptotic gene *BCL2* and inhibition of the apoptotic process. This discovery was the first evidence that microRNAs have a role in the onset of cancer. Following this finding, several studies investigated the role of microRNAs and other small non-coding RNAs in different types of leukemia and cancers in general. This review summarizes the role of small non-coding RNAs in leukemia and describes their possible application in clinical settings as biomarkers and/or targets for therapy.

**Abstract:**

In 2020, more than 60,500 people were diagnosed with leukemia in the USA, and more than 23,000 died. The incidence of leukemia is still rising, and drug resistance development is a serious concern for patients’ wellbeing and survival. In the past two decades, small non-coding RNAs have been studied to evaluate their functions and possible role in cancer pathogenesis. Small non-coding RNAs are short RNA molecules involved in several cellular processes by regulating the expression of genes. An increasing body of evidence collected by many independent studies shows that the expression of these molecules is tissue specific, and that their dysregulation alters the expression of genes involved in tumor development, progression and drug response. Indeed, small non-coding RNAs play a pivotal role in the onset, staging, relapse and drug response of hematological malignancies and cancers in general. These findings strongly suggest that small non-coding RNAs could function as biomarkers and possible targets for therapy. Thus, in this review, we summarize the regulatory mechanisms of small non-coding RNA expression in different types of leukemia and assess their potential clinical implications.

## 1. Background

Leukemia arises as a consequence of the accumulation of underdeveloped or abnormal leukocytes in the blood and bone marrow. Leukemia is classified in a variety of groups traditionally based on clinical presentation (acute or chronic) and cell of origin lineage (lymphocytic or myelogenous) [1]. Acute leukemia arises quickly with a rapid onset of symptoms and is characterized by the accumulation of poorly differentiated blast cells. In contrast, chronic leukemia develops slowly with a gradual onset of symptoms and is characterized by the accumulation of more mature yet dysfunctional cells. Since hematopoietic stem cells generate lymphoid cells (including T cells, B cells and natural killer cells) and myeloid cells (including monocytes, macrophages, neutrophils, basophils, eosinophils, erythrocytes and megakaryocytes), the major subtypes of leukemia are traditionally classified into four classes:

(1) Chronic lymphocytic leukemia (CLL) is the most common human leukemia, characterized by the accumulation of incompetent CD5+ B lymphocytes. Most CLL cases occur in the elderly when mature yet abnormal lymphoid cells undergo hyperplasia, leading to the development of a monoclonal population of dysfunctional lymphocytes [2].
(2) Acute lymphocytic leukemia (ALL) constitutes a family of genetically heterogeneous lymphoid neoplasms derived from B and T lymphoid progenitors [3]. Most ALL cases occur in children, when lymphoid blasts replicate without developing into normal B and T cells [4].
(3) Acute myeloid leukemia (AML) is a heterogeneous clonal disorder characterized by the proliferation of myeloid blasts and bone marrow failure and is the most common acute leukemia in adults [5].
(4) Chronic myeloid leukemia (CML) develops from a clonal myeloproliferative expansion of transformed hematopoietic myeloid cells of monocytic, erythroid and megakaryocytic lineages. Most CML cases occur in people between the ages of 25 and 60 when monoclonal populations of self-renewing, dysfunctional myeloid cells undergo hyperplasia [6].

These malignancies share similarities; however, AML, ALL and chronic lymphoproliferative and myeloproliferative neoplasms are extremely diverse cancers based on cell of origin, biology and clinical management [7]. Indeed, specific chromosomal aberrations lead to the activation of proto-oncogenes or loss of tumor suppressor genes [8,9,10]. A milestone in the field of chromosomal abnormalities in cancer occurred in 1960, with the publication of the first article by Nowell and Hungerford describing the association of CML with the presence of a small-size chromosome, known today as the Philadelphia (Ph) chromosome. The detection of the Philadelphia chromosome established, for the first time, that chromosome abnormalities in leukemia are acquired and thus a hallmark of neoplastic cells [11]. Following this discovery, substantial effort has been made to understand the molecular mechanism driving the development of such malignancies and provide effective treatment [12,13,14]. Remarkably, the study of distinctive chromosomal aberrations in CLL led to the astonishing discovery that microRNA genes (miRNAs) have a key role in the onset and progression of cancer [15]. The first evidence that miRNAs are involved in cancer was published by our group in 2002, describing the loss of *miR-15a/16-1* in CLL [15,16]. In 2005, we showed that *miR-15a/16-1* regulates the expression of *BCL2*, an essential antiapoptotic gene [17]. Since 2002, the study of small non-coding RNAs (ncRNAs) has guided modulation of gene expression in both physiological and pathological processes and laid the groundwork for the development of the most innovative detection tools and targeted therapies [18,19,20]. Indeed, genomic alterations in cancer cells can affect miRNA loci or other ncRNAs, and disruption of such mechanisms can lead to the dysregulation of genes that may promote survival or hinder apoptosis [17,21,22,23,24]. Following the first discoveries of Dr. Croce’s group on miRNA dysregulation in CLL, Lu et al. developed an miRNA microarray to analyze miRNA profiles in samples from cancer patients [25]. The examination of over 300 cancer samples led to the discovery that miRNA expression profiles classify human cancers according to lineage and differentiation state [25]. Later on, other small non-coding RNAs were also found to be involved in cancer [23,26,27,28]. This review reports the story of such discoveries, describes the molecular mechanism of the most significant non-coding genes and revises their most recent applications in the treatment of patients. 

## 2. miRNA Biogenesis

miRNA genes are transcribed by RNA polymerase II into long precursors (pri-miRNA) that are processed by the microprocessor complex (Drosha and DGCR8) into hairpin-shaped RNAs (pre-miR) and exported to the cytoplasm by exportin 5 (EXP5) [29]. In the cytoplasm, pre-miRNAs are cleaved by Dicer and loaded onto Argonaut proteins to form the pre-RISC complex [30]. The duplex structure is unwound, and the seed region at the 5′ ends of the ‘guide’ (mature) miRNA strand is presented to scan for partial complementary sequences on mRNAs’ 3′UTRs [31,32]. The binding of the 3′UTRs of mRNAs to an miRNA seed region causes mRNA degradation and/or inhibits mRNA translation, resulting in decreased levels of gene expression [32]. This mechanism is responsible for fine-tuning the expression of genes, and it is a fundamental process for cells’ commitment, differentiation, response to stimuli and apoptosis [33]. 

## 3. miRNAs in Leukemia 

### 3.1. miRNAs in CLL

B cell chronic lymphocytic leukemia (CLL) is the most common human leukemia in the Western world [2]. CLL can occur in an indolent or aggressive form, both characterized by the accumulation of CD5+ B lymphocytes with specific genetic abnormalities [2]. Prognostic markers such as a high expression of unmutated immunoglobulin heavy variable genes (UM-Ig-VH) and a high level of 70 kD zeta-associated protein (ZAP70) are associated with an unfavorable prognosis [34]. Additionally, chromosomal alterations are identified in more than 80% of cases and are used to stratify patients in risk groups: (i) low risk: normal karyotype or 13q deletion; (ii) intermediate risk: 11q deletion or trisomy 12; and (iii) high risk: 17p deletion or complex karyotype [35,36]. Many CLL cases show discordant prognostic factors [37,38]; thus, the identification of new parameters to establish the clinical outcome and to provide knowledge for the development of new therapeutic strategies is essential. 

The study of chromosomal alteration in CLL led to the discovery that miRNAs have a key role in cancer and could function as biomarkers and possible targets for treatment [15,39] (Table 1). 

**Figure 1:**
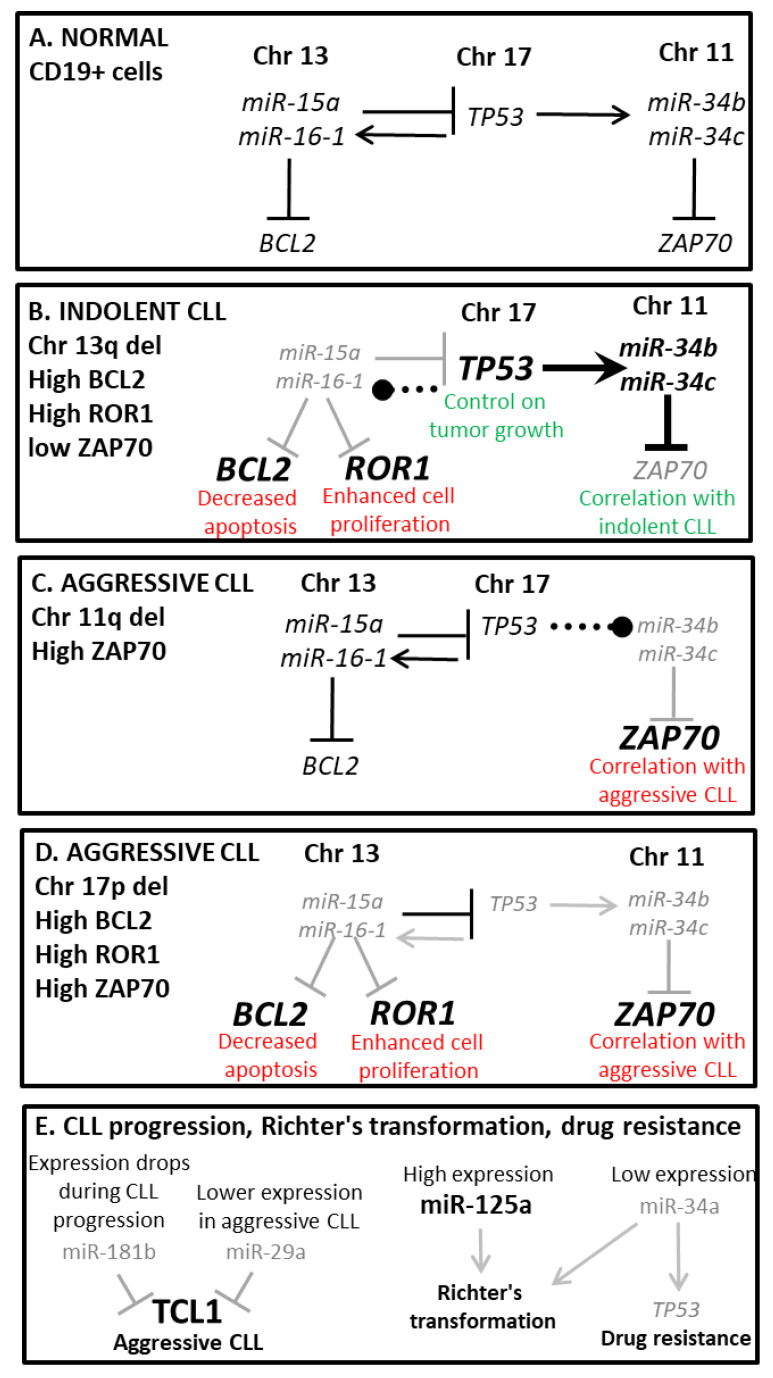
Interplay between microRNA expression and chromosomal aberration in CLL.

This story begins in early 2000, when our group initiated a research project aimed to identify the tumor suppressor gene targeted by the most common genomic aberration in CLL, deletion of the short arm of chromosome 13 [15]. At that time, deletions of the 11q region were known to target the ATM gene [70,71], while deletions of the 17p region were known to encompass the p53 gene [72,73]. These aberrations, often found in association with the deletion of 13q, were recognized predictors of aggressive disease [74]. However, as opposed to the 11q and 17p abnormalities, the 13q deletion did not seem to affect a coding gene. Large deletions of the 13q chromosome were common and found in at least 50% of CLL cases either as a sole abnormality or in combination with other aberrations [35]. Interestingly, patients where 13q was the sole chromosomal abnormality usually showed an indolent disease [35]. In 2002, after extensive analysis, our group provided the first evidence that a non-coding small RNA, known as *miR-15a/16-1*, was the functional target of the 13q14 deletion in CLL [15]. This discovery led our group to investigate *miR-15a/16-1*’s role in CLL, and in 2005, we demonstrated that *miR-15a/16-1* has tumor suppressor functions and can induce apoptosis by targeting the B cell lymphoma 2 (*BCL2*) gene, a potent inhibitor of the apoptotic program [17]. Additionally, we verified that the expressions of *miR-15a/16-1* and Bcl2 in CLL samples from patients are inversely correlated [17]. Furthermore, microarray experiments where samples from CLL patients with high vs. low levels of *miR-15a/16-1* were compared led to the identification of a gene signature containing *MCL1*, another antiapoptotic *BCL2* family member associated with B-CLL cell survival and chemotherapy resistance [75]. These remarkable discoveries revealed that alterations in non-coding genes can lead to malignant transformation of cells, overruling the previous scientific dogma that only protein-coding genes are involved in cancer pathogenesis [76]. Remarkably, in addition to the fact that half of newly diagnosed CLL patients carry a large deletion of the short arm of chromosome 13, mutations, microdeletions and other factors such as allele-specific transcription mechanisms also regulate the expression of miR-15a/16-1 in most CLL patients [16,77]. As a result, about 90% of CLLs show a deficit of *miR-15a/16-1* expression, indicating that the loss of regulation upon the apoptotic mechanism mediated by *miR-15a/16-1* is, in fact, the driver event leading to CLL onset [52]. This discovery was a key element in the design of the Bcl2 inhibitor venetoclax, which was approved by the FDA in 2016 for the treatment of aggressive CLLs [39]. Importantly, our group recently showed that in addition to *BCL2*, *miR-15a/16-1* also targets ROR1, an embryonic oncoprotein that, in postpartum tissues, is only expressed on the membrane of cancer cells [52]. Thus, we tested a humanized anti-ROR1 antibody, cirmtuzumab, to evaluate its effectiveness when used in combination with venetoclax [52]. We found that cirmtuzumab enhances the in vitro cytotoxic activity of venetoclcax for CLL cells with a high-level expression of ROR1, indicating that combining these drugs may have a synergistic effect in CLL patients, by simultaneously targeting ROR1 and Bcl2 in the same leukemic cells [52]. Such a treatment strategy is exceptionally valuable because it targets two driver genes that, within the same cancer cell, are overexpressed as a consequence of the same deficit, i.e., the loss of *miR-15a/16-1*, thus preventing the possibility of selecting a resistant clone [52]. In addition, we investigated the interactions between *miR-15a/16-1* and other chromosomal alterations observed in CLL, to evaluate what miRNA-mediated molecular pathway could explain the prognostic implications of 11q, 17p and 13q deletions in CLL [69]. Interestingly, the 11q deleted region includes the *miR-34b/c* cluster locus. Binding sites for p53 were found upstream the *miR-15a/16-1* locus on chromosome 13, and upstream the *miR-34b/c* locus on chromosome 11 [69]. In 2011, we published a study showing that p53 induces the expression of *miR-15a/16-1* and *miR-34b/c*, while *miR-15a/16-1* targets *TP53*, and *miR-34b/c* targets *ZAP-70*, an indicator of an unfavorable prognosis [69]. Thus, in patients with 13q deletion as a sole abnormality, the loss of *miR-15a/16-1* expression leads to higher levels of pro-apoptotic p53, explaining the association of 13q- with indolent disease. Indeed, in this scenario, the number of apoptotic cells decreases because of the higher level of Bcl2, but the intact p53 pathway keeps the tumor growth relatively low and leads to transactivation of *miR-34b/c*, with a consequent low ZAP-70 level [69] (Figure 1A,B). In CLL patients with 11q deletion, since *miR-15a/16-1* is not deleted, *TP53* is not upregulated. In this scenario, lower control on apoptosis is provided, explaining the more aggressive presentation. Furthermore, p53-driven transactivation of *miR-34b/c* is ineffective, since this miRNA is deleted, leading to a higher expression of *ZAP-70* [69] (Figure 1C). Lastly, deletion of 17p and *TP53* mutations highly correlates with poor outcomes and response to treatment [78] (Figure 1D). However, more than half of the refractory cases cannot be explained by the lack of p53 expression alone [79]. A study carried out by Zenz et al. showed that *miR-34a* expression is lower in chemotherapy-resistant CLL regardless of the 17p deletion/*TP53* mutation [66]. Thus, in addition to *miR-15a/16-1*, other miRNAs have a role in CLL development and progression, such as the *miR-34b/c* cluster and *miR-34a*. Interestingly, we found *miR-34a* to be downregulated in CLL patients that undergo Richter transformation, a rare but serious complication that affects about 5% of CLL patients and is characterized by the sudden development of an aggressive large diffuse B cell lymphoma with a poor prognosis [42]. We established that downregulation of *miR-34a* and overexpression of *miR-125a* can predict the transformation to lymphoma in more than 50% of CLL patients up to 5 years prior, with a false positive rate of less than 10%. This is a significant discovery because no marker is currently available to assign a risk factor for Richter transformation to patients [42] (Figure 1E). Since several microarray experiments revealed miRNA signatures in CLL [25,80], we studied the dysregulation of several other miRNAs in CLL onset, progression and resistance development. Among the most dysregulated miRNAs, the expression of *miR-181b* was investigated by Visone et al., in the progression from a clinically indolent disease to a more aggressive form [61]. In this study, we showed that, when compared to normal B cells, *miR-181b* is downregulated in CLL cells form both indolent and aggressive cases, and that *miR-181b* expression diminishes while the disease progresses from an indolent to an aggressive stage. This significant discovery suggests that *miR-181b* could be used as a biomarker to track disease progression and, potentially, as a treatment agent to reduce expansion of B-CLL leukemic cells [81,82]. As opposed to *miR-181*, *miR-29* was found overexpressed in CLL cells from both indolent and aggressive cases when compared to normal B cells [63]. However, similar to *miR-181b*, indolent cases show a higher expression than aggressive cases [16]. Interestingly, both *miR-181b* and *miR-29* regulate the expression of the T cell leukemia 1A gene, *TCL1A*, which is highly expressed in patients with aggressive disease, suggesting that their downregulation may contribute to disease staging by driving the overexpression of Tcl1 [18] (Figure 1E). Since *TCL1* overexpression is a contributing event in the pathogenesis of the aggressive form of CLL, we sought to identify other miRNAs targeting *TCL1* that could play a role in the development and progression of CLL. We found that *miR-3676* targets a region of the 3′UTR of *TCL1* containing three consecutive 28 bp repeats [24]. While studying *miR-3676*, we realized that it is embedded in a tRNA sequence. Further analysis showed that *miR-3676* is a tRNA-derived small RNA (tsRNAs), and we re-named this molecule *ts-53* [26]. tsRNAs are single-stranded unique sequences of 16–48 nucleotides ending with a stretch of 3 or more Ts and produced in the nucleus as a consequence of the 3′-end cut of a tRNA precursor performed by the enzyme RNase Z [83]. We demonstrated that *ts-53* and its neighbor *ts-101* (previously known as *miR-3676* and *miR-4521*, respectively) interact with both Ago and Piwi proteins; thus, they could act both as miRNAs (which function as post-transcriptional silencers of target genes) or as piRNAs (which function as epigenetic silencers of transposons and other genetic elements) [23]. Since the genes codifying for these tsRNAs are located in tandem within the region of chromosome 17 that is deleted in 17p- CLL, *ts-53* is co-deleted with TP53 in 17p- CLL cells [24]. In addition, we found that *ts-53* is also downregulated in all CLL groups [24,84]. Remarkably, *ts-53* targets the 3′UTR of *TCL1*, and its downregulation in CLL is inversely correlated with *TCL1* expression, suggesting that tsRNAs can control gene expression post-transcriptionally [24]. Additionally, the *ts-53* and *ts-101* expression pattern is positively correlated with *ZAP-70* methylation, and the *ts-101* sequence shows a complementarity of 13 out of 14 bases to the *ZAP-70* promoter region [23]. This evidence supports the hypothesis that tsRNAs could interfere with the epigenetic regulation of genes by providing sequence specificity for the Piwi-ribonucleoprotein complexes to interact with the promoter of the target gene [85]. Thus, similar to our previous work on *miR-15a/16-1* located at 13q, we demonstrated that *ts-53* can be inactivated in CLL by several mechanisms such as deletions, mutations and processing defects. These data show that small non-coding RNAs of different types can affect the onset and progression of CLL and possibly other cancers and may represent valuable diagnostic tools and targets for therapy [85,86]. 

### 3.2. miRNAs in ALL

Acute lymphocytic leukemia (ALL) is the most common type of childhood leukemia, developing from B or T cells at different stages of maturity [4,87]. ALL also affects adults, with a significantly poorer response to treatment and survival [88,89]. In 2008, the World Health Organization classified ALL into two major groups, based on lineage: B lymphoblastic and T lymphoblastic leukemia. In addition, B lymphoblastic leukemia is divided into two subtypes: B-ALL with recurrent genetic abnormalities and B-ALL not otherwise specified. B-ALL with recurrent genetic abnormalities is further delineated based on specific chromosomal rearrangements [90]. B cell ALL accounts for ~75% of cases, while T cell ALL constitutes the remaining cases [91]. 

In 2009, Schotte et al. cloned 105 known and 8 new miRNA genes expressed in B-ALL and T-ALL. This study revealed ALL subtype-specific miRNA profiles [92]. Indeed, this report shows that the expression of miRNA genes varies in different subtypes of pediatric ALL and normal CD34b progenitor cells and suggests that the differential expression of specific miRNAs, such as *miR-196b* and *miR-708*, is more associated with the leukemic subtype than with the maturation status of cells [92]. In addition, miRNA expression profiling can discriminate childhood T- from B-acute lymphoblastic leukemia [93] and cluster acute leukemia samples into three groups corresponding to specific chromosomal rearrangements: (i) *BCR-ABL*, a hallmark of CML also found in high-risk ALL; (ii) t(12;21)(p13;q22), also known as *ETV6/RUNX1* or *TEL-AML1* translocation, which is associated with a good outcome; and (iii) *MLL1* disruption on chromosome 11q23, which correlates with a poor outcome [94,95,96,97]. Thus, miRNA patterns can distinguish B-lineage from T-lineage ALL and classify ALL of different cytogenetic groups. In 2012, de Oliveira et al. noted that leukemic blasts showed lower expression levels of *miR-100*, *miR-196b* and *let-7e* when compared to normal bone marrow cells (BMCs) [40]. Conversely, when clustering patients according to their biological features, an increased expression of *miR-100* appeared associated with the t(12;21) translocation. This observation suggests the possibility of a t(12;21)-specific regulation of *miR-100* [40] in *ETV6/RUNX1* ALL, which shows better prognostic outcomes when compared to the other groups. Shortly after, Li et al. showed that lower expressions of *miR-100* and *miR-99a* are associated with a poor prognosis and shorter survival in ALL. Moreover, this report shows that these miRs inhibit the expression of *IGF1R* and *mTOR* and their downstream oncogene *MCL1* [41]. In 2015, Yang et al. [52] identified *miR-181a* as the most significantly downregulated miRNA in the peripheral blood of childhood ALL patients with the t(12;21) translocation compared to precursor B-ALL [57]. This report shows that *miR-181*a and *ETV6/RUNX1* regulate each other. Thus, a double negative loop involving *miR-181*a may contribute to the *ETV6/RUNX1*-driven arrest of differentiation in pre-B ALL and suggests that *miR-181a* is a lost tumor suppressor in ALL [58]. In support of this hypothesis, in 2017, Nabhan et al. noted a decrease in the expression level of *miR-181a* in the serum of children diagnosed with ALL [59]. Conversely, Verducci et al. proposed that *miR-181a* is an onco-miRNA in ALL, and that its biological effects are mediated by *EGR1* in Jurkat cell line models [98]. Shortly after, Haque et al. suggested that *miR-181a* could be an assimilated oncomiR in ALL via exosome-mediated uptake [60]. Since exosomes from patients showed overexpression of *miR-181a* when compared to exosomes from healthy donor controls, in this study, exosomes isolated from the serum of ALL patients were used to condition the medium of leukemic B cell lines. This conditioning promoted cell proliferation of leukemic cell lines by suppressing pro-apoptotic genes such as *BAD* and *BAX* and by upregulating proliferative and pro-survival genes such as *PCNA, Ki-67, MCL-1* and *BCL2* [60]. In addition, the authors carried out silencing of exosomal *miR-181a* using an *miR-181a* inhibitor and confirmed that *miR-181a* inhibitor treatment reverses the exosome-induced leukemic cell proliferation in vitro [60]. Altogether, these results indicate that *miR-181* is involved in ALL, but it is still unclear whether its role is that of a tumor suppressor or an oncomiR. Other studies showed additional dysregulated miRNAs in ALL. In 2013, Gimenes-Teixeira found that *miR-221* and *miR-374* were expressed at significantly higher levels in CD56+ T-ALL than in CD56- T-ALL, and in leukemic blasts compared to normal thymocytes and normal T cells. In addition, high expression of *miR-221* was associated with a poorer prognosis [62]. In 2018, Swellam et al. investigated the expression signature of *miR-125b-1* and *miR-203* among childhood ALL cases and proposed these miRNAs as molecular markers for the diagnosis of childhood ALL. They noticed that when compared to control samples, newly diagnosed children with ALL show a significantly higher expression level of *miR-125b-1* and a significantly lower expression level of *miR-203* [43]. Moreover, *miR-125-1* was increased in T-ALL compared to other ALLs. Thus, miRNAs are involved in ALL development and staging and could represent excellent diagnostic tools or targets for therapy.

### 3.3. miRNA Signatures in AML

Acute myeloid leukemia is the most common acute leukemia in adults and is characterized by the abnormal proliferation of myeloid stem cells [99]. AML often evolves from myelodysplastic syndromes (MDSs), a very heterogeneous group of myeloid disorders [100]. Cytogenetic and molecular findings provided a model of stepwise genetic progression that may explain the development and evolution of MDSs to AML [101]. However, while the classification for AML is based on cytogenetic and genetic abnormalities, the classification for MDSs/AML still relies on morphological findings [102]. Genetic mutations are identified in more than 97% of AML cases [103], and cytogenetic abnormalities are detected in 50% to 60% of newly diagnosed AML cases [104,105]. Specifically, t(8:21) translocations resulting in the formation of the chimeric protein RUNX1-RUNX1T1 are generally associated with a favorable prognosis. However, a high frequency of relapse is observed in pediatric cases showing a specific epigenetic signature [106]. Rearrangements of chromosome 11 involving the *MLL* gene are more common in pediatric cases and are associated with an intermediate prognosis [107]. The t(15;17) translocation, resulting in the expression of the PML-RARα oncoprotein, is found in almost all acute promyelocytic leukemias (APLs), a very aggressive subtype of AML [108].

**Figure 2:**
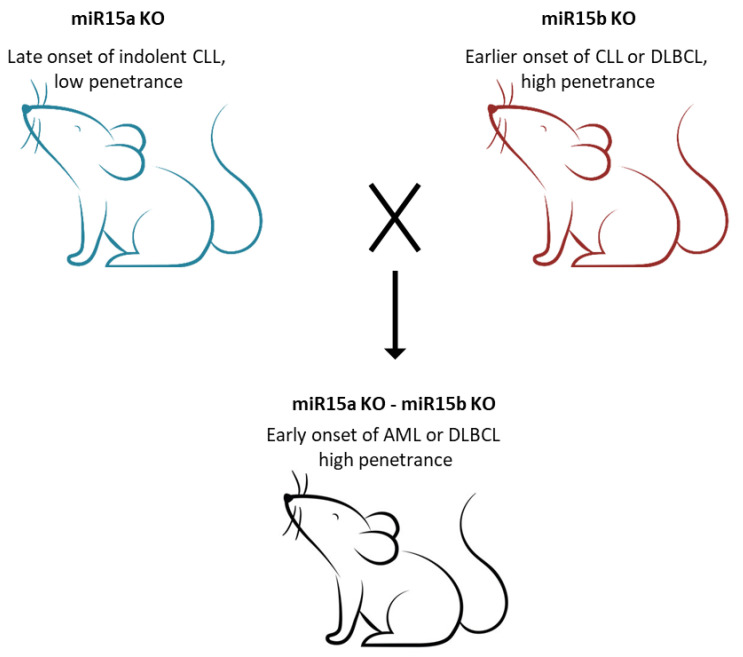
Double KO mice, lacking both miR15a and miR-15b, develop AML.

Following the data obtained from microarray experiments carried out on AML samples from patients, miRNA dysregulation was shown in AML. A specific signature of 26 miRNAs was identified in AML, associating with specific AML karyotype subgroups [109]. Shortly after, *miR-224*, *miR-368* and *miR-382* were found almost exclusively overexpressed in t(15;17) AMLs, whereas the *miR-17-92* cluster was found overexpressed in patients with MLL rearrangements [56]. Dysregulation and mutations of *miR-29* family members were also reported to play a role in AML progression and pathogenesis [64,65]. In addition, *miR-29b* targets DNA methyltransferase including *DNMT3A*, *DNMT3B* and *Sp1* (a transcriptional regulator of *DNMT1*) [110]. Thus, downregulation of *miR-29b* promotes DNA hypermethylation and gene silencing. In addition, *miR-29a* and *miR-29b* alter the expression of genes involved in apoptosis, cell cycle progression and cellular proliferation, by affecting the AKT pathway and the phosphorylation of Rb. The study which reported such a finding suggested that *miR-29a* and *miR-29b* may be considered as therapeutic targets in AML [111]. The expression of *miR-34* cluster members was also studied in AML. In 2010, Pulikkan et al. showed that *miR-34a* blocks myeloid proliferation by targeting *E2F3* during granulopoiesis, but it is downregulated in AML with mutations affecting the CCAAT enhancer binding protein alpha (*C/EBPα*). *C/EBPα* is a myeloid tumor suppressor and a key regulator of granulopoiesis. Importantly, *C/EBPα* directly regulates *miR-34a* during granulopoiesis. Thus, in AML where *C/EBPα* function is lost, *miR-34a* is blocked, leading to *E2F3* upregulation, which results in increased proliferation of myeloid progenitors [112]. Interestingly, another study reported that in complex karyotype AML, the role of *miR-34a* in clinical prognosis is influenced by the status of p53. Indeed, in complex karyotype AML patients with functional *TP53*, upregulation of *miR-34a* associates with shorter overall survival, whereas in patients with impaired *TP53*, it associates with longer overall survival and chemotherapy sensitivity [67]. Interestingly, no direct correlation between p53 pathway genes and *miR-*34a expression was found, suggesting that the induction of *miR-34a* may be p53 independent [67]. *MiR-181* was also evaluated in AML, and its overexpression was positively associated with a good clinical outcome [68,113]. Interestingly, *miR-181* family members were found upregulated in patients with *C/EBPα* mutations, as opposed to *miR-34a*. This observation suggests that microRNAs have a complex effect on the myeloid differentiation pathway [114]. In cytogenetically normal AML patients, *miR-181* contributed to a better clinical outcome by regulating Toll-like receptors and interleukin-1β, while in cytogenetically abnormal AML, *miR-181* contributed to a better clinical outcome by regulating *HOXA7*, *HOXA9*, *HOXA11* and *PBX3* [115]. Moreover, *miR-181b* can increase AML drug sensitivity through downregulation of *HMGB1* and *MCL-1*, and it is indeed downregulated in relapsed and refractory AML patients [116]. Recently, our group discovered that *miR-15/16* genes have an important role in AML development [53]. A member of the *miR-15/16* family (*miR-15b/16-2* cluster) is located at 3q25 and is almost identical to *miR-15a/16-1*. Since the tumor suppressor function of the *miR-15a/16-1* cluster was well established in CLL, we sought to determine whether *miR-15b/16-2* could have a role in cancer and generated *miR-15b/16-2* knockout mice [54]. Interestingly, we noted that by the age of 15–18 months, *miR-15b/16-2* KO mice developed a CD5+ B cell proliferation similar to human CLL, with a penetrance of 60%. However, the expression of *BCL2* was found to be only mildly upregulated, whereas a robust upregulation of Cyclin D1 and Cyclin D2, which are predicted targets of *miR-15/16*, was revealed [54]. Following these exciting results, we decided to study the effect of the combined loss of both loci and generated a double KO mouse model by crossbreeding *miR-15a/16-1* and *miR-15b/16-2* KO mice [55]. Surprisingly, the deletion of both clusters promoted myeloproliferative disorders in the majority of the mice by the age of 5 months, suggesting that a fraction of AML could be caused by the loss of both *miR-15/16* clusters [55] (Figure 2). In addition, we observed a striking upregulation of Cyclin D1, Cyclin D2 and Bcl-2 [55]. These astonishing results led us to compare the expression of the two *miR-15/16* clusters in patients with myelodysplastic syndrome (MDS) and AML patients [53]. We discovered that reduced expression of *miR-15a*, *miR-15b* and *miR-16* can predict the progression from MDS to AML, thus suggesting that the expression of these two *miR-15/16* clusters can be a valuable marker to stratify AML patients for therapy [53]. Moreover, we discovered that, while ~79% of AML patients show a downregulation of either *miR-15a/16-1* or *miR-15b/16-2*, ~21% of patients show a downregulation of both, suggesting that loss of expression of both *miR-15a/16-1* and *miR-15b/16-2* is a common event in AML [53]. Thus, the fraction of AML cases that lost the expression of *miR-15/16* should be treated with venetoclax, alone or in combination with anti-ROR1 monoclonal antibodies. Treatment with venetoclax should also be considered to prevent the transition of MDS to AML. Indeed, venetoclax has very mild side effects and shows remarkable activity in AML.

### 3.4. miRNA Signatures in CML

Chronic myeloid leukemia (CML) was the first human malignancy to be associated with a chromosomal rearrangement: a translocation involving chromosomes 9q34 and 22q11 [117]. This rearrangement generates the Philadelphia chromosome, encoding for the brc-abl oncoprotein. brc-abl is an active tyrosine kinase that promotes cell growth and replication and is the main driver of CML pathogenesis [118,119]. The natural course of CML begins with a chronic phase (CP) that progresses through an accelerated phase (AP) to the blast crisis (BC) phase [120], a major challenge in the management of CML. The progression to BC is the consequence of continued *BCR-ABL* activity leading to genetic instability, DNA damage and impaired DNA repair [121]. Importantly, the discovery of the Philadelphia chromosome, and the brc-abl oncoprotein, led to the development of the first signal transduction inhibitor (STI) used in a clinical setting, imatinib, a drug designed to directly bind the brc-abl oncoprotein and inhibit its oncogenic activity [122,123]. The introduction of such a drug significantly improved the treatment outcome of CML as brc-abl expression can be reduced by imatinib to very low or nondetectable levels in the majority of patients [124]. However, a variety of mutations are associated with progression to BC, and such mutations in late CP often lead to imatinib resistance. Despite major clinical advances, imatinib resistance is a challenging problem in the management of CML [125].

miRNAs have been demonstrated to play a critical role in the pathogenesis of CML [126]. Indeed, miRNA signatures were described to discriminate CML cells from normal cells [127], to identify responder and non-responder patients [128] and to define clinical phases of CML [45]. In addition, while miRNAs can target and regulate the expression of *BCR-ABL* [46,47], brc-abl can regulate the expression of miRNAs [48]. In 2008, *miR-203* was found hypermethylated in CML. This study showed that *miR-203* targets ABL1 and the bcr-abl fusion protein, thus preventing tumor cell proliferation. Hence, *miR-203* may function as a tumor suppressor, and restoration of its expression might have therapeutic benefits in CML [49]. In 2018, a *BCR-ABL* loop involving MYC and *miR-150* was described showing that bcr-abl inhibits *miR-150* expression in CML cells via the transcriptional activation of *MYC* and its simultaneous recruitment to a specific locus of the *miR-150* gene, where myc binds and acts as a direct repressor of *miR-150* transcription [50]. Most Philadelphia chromosome-positive CMLs are treated with bcr-abl kinase inhibitors such as imatinib or dasatinib, or other tyrosine kinase inhibitors (TKIs) that prevent the interaction between bcr-abl and ATP. Nevertheless, ∼2% of patients become resistant to TKIs, and in such cases, allogeneic hematopoietic stem cell transplantation (allo-HSCT) is the only curative treatment for CML [51]. For these reasons, several studies were carried out and found that the dysregulation of miRs and their predicted target genes is different in CML phases and after treatment with TK inhibitors [128]. Indeed, imatinib treatment leads to increased expression of *miR-150* and *miR-146a*, and reduced expression of *miR-142-3p* and *miR-199b-5p* [44]. Interestingly, both *miR-150* and *miR-146a* are regulated by BCR-ABL1 [48,50], and *miR-150* may be a useful biomarker for disease progression, where its lower expression correlates with a poor prognosis and more advanced phases of CML [44,45]. Altogether, these studies suggest that TKI may be able to restore the levels of some miRNAs, and that this process may have a role in mediating the effect of TKIs on CML survival and apoptosis.

In addition to TKI resistance, another important event that may be associated with miRNA dysregulation in CML is the transition from the chronic phase to blast crisis. The development of BC is the consequence of sustained *BCR-ABL* activity [120], which leads to genomic instability and DNA damage [120]. However, the molecular mechanisms responsible for this process are not fully understood. Since we previously showed that loss of *miR-15/16* loci is a key event in the development of AML [55] and CLL [15], we evaluated the correlation between the expression of *miR-15a/16* and the transition of CML from the chronic phase to the blastic phase [129]. We collected samples from patients in the CP, samples from patients in BC and sequential samples from patients where the first set was obtained during the CP and the second set during BC. We found that CMLs in the CP express less *miR-15a, miR-15b* and *miR-16* than normal CD34+, and that CMLs in BC express less *miR-*15a, *miR-*15b and *miR-16* than CMLs in the CP. Thus, we analyzed paired samples collected from the same patients during the chronic phase and blast crisis and found a significant decrease in the expression of all these miRs during the progression of the disease. In addition, we observed a significant overexpression of their targets, Bmi-1, ROR1 and Bcl-2. Thus, loss of both *miR-15/16* clusters is a driving event in the transition from the chronic phase to blast crisis [129]. CML progression from the CP to BC is likely mediated by a progressive downregulation of *miR-15/16,* resulting in the overexpression of Bcl-2, ROR1 and Bmi-1. Therefore, as for CLL, targeting different oncogenes activated by the same genetic/epigenetic alteration may be a more successful treatment approach, with the considerable advantage of tackling the problem of resistance development [129].

## 4. Conclusions

The study of small non-coding RNAs has extraordinary implications in the management of cancer patients. The identification of new markers for differential diagnosis can improve the stratification of patient for the most effective treatment. In addition, small non-coding RNAs can be exploited to monitor disease progression and drug resistance onset. The study of small non-coding RNAs is also crucial for the discovery of new targets for therapy. Indeed, the development of novel drugs to use in combination for synergistic effects is essential for the design of therapeutic strategies that can prevent drug resistance onset. While investigating the role of genetic abnormalities in CLL, our group revealed that *miR-15a/16-1* is lost in CLL [15], and that these molecules regulate the apoptotic process by targeting Bcl2 [17]. This observation led to the groundbreaking discovery that microRNAs have a key role in the fine-tuning of gene expression and affect cell survival and apoptosis. The identification of the *BCL2* translocation in 1984 [130] and the description of the *miR-15a/16-1*-mediated post-transcriptional mechanism of regulation of Bcl2 in 2005 [17] were fundamental steps for the development of one of the most effective drugs for the treatment of CLL, venetoclax. In addition, we found that *miR-15a/16-1* targets ROR1, an onco-embryonic surface protein expressed on CLL cells but not on normal cells [21]. The anti-ROR1 monoclonal antibody cirmtuzumab is currently being evaluated for treatment, and we showed that it has a synergistic effect with the in vitro cytotoxic activity of venetoclcax [21]. Remarkably, *miR-15a/16-1* is often downregulated in other hematopoietic malignancies and cancers of different types. Indeed, most AML patients show a downregulation of either *miR-15a/16-1* or *miR-15b/16-2,* and ~21% of AML patients show a downregulation of both [53]. These AML patients should be considered for treatment with venetoclax, alone or in combination with cirmtuzumab. We also found a correlation between the expression of *miR-15a/16* and the transition of CML from the chronic phase to the blastic phase [129], showing that the expression of these miRs decreases during the progression of the disease, while their targets are overexpressed. Thus, loss of both *miR-15/16* clusters is a driving event in the transition from the chronic phase to blast crisis, and CML patients should also be considered for combination therapy with venetoclax and cirmtuzumab. Other microRNAs have been found dysregulated in leukemia of different types and represent markers for disease classification, progression and treatment response. For instance, the *miR-181* family members are dysregulated in several types of leukemia. Downregulation of *miR-181b* is a potent indicator of disease progression in CLL patients [61], and *miR-181*b has been studied as a possible therapeutic agent for CLL [82]. In addition, the same microRNA is downregulated in relapsed and refractory AML patients [116]. *miR-181a* seems to be involved in ALL; however, it is still unclear whether its role is that of a tumor suppressor or an oncomiR [57,60]. These studies suggest that dysregulation of *miR-181* family members may be exploited for the development of diagnostic tools and therapy. Downregulation of *miR-34a* and upregulation of *miR-125a* can predict Richter’s transformation of CLL [42], and signatures of microRNAs have been detected in ALL discriminating lineages and cytogenetic groups [93,96]. In addition, feedback loops have been identified between microRNA genes and protein-coding genes: TP53 transactivates *miR-15a/16-1* and *miR-34b/c* in CLL [69], and MYC binds and directly inhibits *miR-150* transcription in CML [50]. The study of microRNAs in cancer has greatly improved our understanding of cancer pathogenesis and opened new possibilities for the development of superior diagnostic tools and targets for therapy. In addition, we have discovered that other small non-coding RNAs are dysregulated in cancer, such as tRNA-derived small RNAs, tsRNAs [23]. The dysregulation of these molecules and their role in the fine-tuning of genes can also be exploited to improve the array of diagnostic tools for accurate differential diagnosis, and to provide new targets for the development of more specific anticancer drugs. In conclusion, the study of small non-coding RNAs has greatly improved our understanding of cancer pathogenesis and opened new possibilities for the development of superior diagnostic tools and targets for therapy. Small non-coding RNAs are key regulators of gene expression, and the study of their role in cancer onset, progression, relapse and resistance to therapy is essential to improve the stratification of patients for successful treatment, and to lay the groundwork for the design of the next generation of anticancer drugs.

## Figures and Tables

**Table 1:** Differentially expressed small non-coding RNAs in chronic and acute human leukemias.

miRNA	Expression	Biomarker	Reference
let-7e	lower in ALL blast vs. BMC	lower expression associates with poor prognosis in ALL	[40]
miR-100	lower in ALL blast vs. BMC, higher in ALL with t(12;21) vs. other groups	lower expression associates with poor prognosis in ALL	[40,41]
miR-125a	higher in CLL patients that develop Richter’s syndome	biomarker for Richter trasformation in CLL	[42]
miR-125b-1	higher in newly diagnosed childhood ALL vs. controls, higher in T-ALL vs. other ALLs	biomarker for ALL	[43]
miR-142-3p	lowered by imatinib treatment in CML	biomarker for TKI response in CML	[44]
miR-146a	increased by imatinib treatment in CML	biomarker for TKI response in CML	[44]
miR-150	increased by imatinib treatment in CML	biomarker for TKI response in CML, lower expression associates with poor prognosis and staging	[44,45,46,47,48,49,50,51]
miR-15a/16-1	deleted in most CLL cases, lower in AML vs. MDS and controls	driver event in CLL onset, interacts with other chromosomal alterations in CLL prognosis, biomarker for sensitivity to venetoclax	[15,16,17,39,52,53,54,55]
miR-15b/16-2	lower in AML vs. MDS and controls	promotes B cell malignancies, driver event in AML pathogenesis, predicts progression from MDS to AML	[53,54,55]
miR-17-92	overexpressed in AML patients with MLL rearrangements		[56]
miR-181a	lower in ALL with t(12,21) vs. pre-B ALL, higher in exosomes from ALL vs. heathy donors	involved in ALL pathogenesis	[57,58,59,60]
miR-181b	drops during CLL progerssion and is lower in relapsed/refractory AML	marker for CLL progression	[53,61]
miR-196b	lower in ALL blast vs. BMC	biomarker for ALL	[40]
miR-199b-5p	lowered by imatinib treatment in CML	biomarker for TKI response in CML	[44]
miR-203	lower in newly diagnosed childhood ALL vs. controls	biomarker for ALL	[43]
miR-221	higher in DC56+T-ALL	high expression associates with poor prognosis	[62]
miR-224	overexpressed in t(15;17) AMLs		[56]
miR-29a/b	overexpressed in all CLL vs. normal B cells and in idolent vs. aggressive CLL, dysregulated/mutated in AML	marker for CLL staging	[18,63,64,65]
miR-34a	lower in refractory CLL and in CLL patients that develop Richter’s syndome, lower in AML with lost C/EBPα	biomarker for Richter trasformation in CLL and resistance development	[42,66,67,68]
miR-34b/c	lost in 11q- CLL	targets ZAP70, a prognostic marker in CLL	[69]
miR-3676 or ts-53	downregulated in all CLLs vs. controls and co-deleted with TP53 in 17p CLL	belongs to the tRNA-derived small RNA family (tsRNAs), a new family of cancer biomarkers	[24,26]
miR-368	overexpressed in t(15;17) AMLs		[56]
miR-374	higher in DC56+T-ALL		[62]
miR-382	overexpressed in t(15;17) AMLs		[56]
miR-4521 or ts-101	downregulated in all CLLs vs. controls and co-deleted with TP53 in 17p CLL	belongs to the tRNA-derived small RNA family (tsRNAs), a new family of cancer biomarkers	[24,26]
miR-99a	lower expression is associated with poor prognosis and shorter survival in ALL	ALL staging	[41]
